# A Novel Gel-Forming Solution Based on PEG-DSPE/Solutol HS 15 Mixed Micelles and Gellan Gum for Ophthalmic Delivery of Curcumin

**DOI:** 10.3390/molecules25010081

**Published:** 2019-12-24

**Authors:** Na Sai, Xiaoxv Dong, Pingqing Huang, Longtai You, Chunjing Yang, Yi Liu, Wenping Wang, Huimin Wu, Yingchao Yu, Yuanyuan Du, Xin Leng, Xingbin Yin, Changhai Qu, Jian Ni

**Affiliations:** 1 School of Chinese Materia Medica, Beijing University of Chinese Medicine, Beijing 102488, China; yxsaina@126.com (N.S.); dxiaoxv@163.com (X.D.); pqhuang@126.com (P.H.); ylt_svip@163.com (L.Y.); 17810257751@163.com (C.Y.); 18765804002@163.com (Y.L.); wangwenp6@163.com (W.W.); 18811385368@163.com (H.W.); yyc_zwq@163.com (Y.Y.); dyy9401@163.com (Y.D.); lengx11@126.com (X.L.); xb_yin@163.com (X.Y.); 2 School of pharmacy, Inner Mongolia Medical University, Hohhot 010110, China

**Keywords:** curcumin, mixed micelles, PEG-DSPE, Solutol HS 15, gellan gum, ion-sensitive in situ gels, corneal permeation

## Abstract

Curcumin (Cur) is a naturally hydrophobic polyphenol with potential pharmacological properties. However, the poor aqueous solubility and low bioavailability of curcumin limits its ocular administration. Thus, the aim of this study was to prepare a mixed micelle in situ gelling system of curcumin (Cur-MM-ISG) for ophthalmic drug delivery. The curcumin mixed micelles (Cur-MMs) were prepared via the solvent evaporation method, after which they were incorporated into gellan gum gels. Characterization tests showed that Cur-MMs were small in size and spherical in shape, with a low critical micelle concentration. Compared with free curcumin, Cur-MMs improved the solubility and stability of curcumin significantly. The ex vivo penetration study revealed that Cur-MMs could penetrate the rabbit cornea more efficiently than the free curcumin. After dispersing the micelles in the gellan gum solution at a ratio of 1:1 (*v*/*v*), a transparent Cur-MM-ISG with the characteristics of a pseudoplastic fluid was formed. No obvious irritations were observed in the rabbit eyes after ocular instillation of Cur-MM-ISG. Moreover, Cur-MM-ISG showed a longer retention time on the corneal surface when compared to Cur-MMs using the fluorescein sodium labeling method. These findings indicate that biocompatible Cur-MM-ISG has great potential in ophthalmic drug therapy.

## 1. Introduction

The topical application of eye drops is still the most common and well-accepted approach for the treatment of ocular diseases. Conventional pharmaceutical formulations, such as solutions and suspensions, account for nearly 90% of the currently available marketed formulations because of their simplicity and high acceptance by patients [1]. However, one of the major drawbacks associated with topical ocular drug delivery is the rapid and extensive precorneal loss caused by drainage and high tear fluid turnover [2]. Typically, less than 5% of the applied drug penetrates through the cornea/sclera, with intact drug activity at the target tissues, and most of the applied dose often gets absorbed through the nasolacrimal route [3]. In addition, a major part of the drug absorbed systemically results in undesirable side effects.

In order to overcome the disadvantages associated with conventional eye drops, many ocular drug delivery systems have been recently formulated and evaluated. These systems include polymeric micelles, liposomes, dendrimers, oil-water lipid emulsions, and nanoparticles. These delivery systems improve the ocular bioavailability of drugs and reduce severe adverse effects [4]. The improvement of corneal penetration and the prolongation of precorneal residence time are the main strategies applied to overcome precorneal restraints [5]. Studies have shown that polymer micelles improve the ocular bioavailability of poorly water-soluble drugs via the enhancement of their solubility and permeability based on amphiphilic molecules or block copolymers [6]. In situ gel-forming systems (comprising thermosensitive gels, pH-sensitive gels, and ion-sensitive gels) are viscous liquids used to enhance precorneal retention time. They are capable of transitioning to a gel phase upon exposure to physiological conditions. Ion-activated in situ gelling systems have great potential as ocular drug delivery systems due to the presence of mono- and divalent cations, such as Na^+^, K^+^, Mg^2+^, and Ca^2+^, in the tears of the eye [7]. To exploit the benefits of these two dosage forms, we proposed polymeric micelles incorporated in situ ocular gel for ophthalmic drug delivery.

Curcumin (Cur) is a naturally hydrophobic polyphenol and a bioactive component of the herb *Curcuma longa* L. (turmeric). Emerging evidence suggests that curcumin exhibits many pharmacological effects, such as anti-cancer, anti-inflammatory, antimicrobial, antidiabetic, antioxidant, neuroprotective, and hepatoprotective properties [8]. These pharmacological properties form the basis for its application in the treatment of various diseases, including allergies, asthma, bronchial hyperactivity, Alzheimer’s disease, liver fibrosis, psoriasis, Type 2 diabetes, growth disorders, and several types of cancer [9]. Moreover, studies have demonstrated that curcumin is a potent therapeutic candidate for eye diseases, including dry eye disease, corneal neovascularization, conjunctivitis, pterygium, anterior uveitis, glaucoma, and cataracts [10,11]. However, the poor aqueous solubility of curcumin is a limiting factor in its use in the formulation of solutions intended for ocular administration.

In the current study, a novel ophthalmic gel-forming solution based on 1,2-distearoyl-sn-glycero-3-phosphoethanolamine-*N*-[methoxy(polyethylene glycol)-2000] (PEG-DSPE)/ polyoxyethylene esters of 12-hydroxystearic acid (Solutol HS 15) mixed micelle and gellan gum was constructed in order to enhance the solubility, stability, and permeability of curcumin. Moreover, the fluorescent marker coumarin-6 (C6) was used to visualize the transport of mixed micelles to the deep cornea. The results demonstrated that the gel-forming solution was non-irritating and had good gelling ability. After application as eye drops, this gel persisted in the ocular surface for a long time and had good corneal permeability. To our knowledge, this is the first report of an in situ gel system based on PEG-DSPE/Solutol HS 15 mixed micelles and gellan gum for ophthalmic delivery of curcumin.

## 2. Results

### 2.1. Characterization of Curcumin Mixed Micelles (Cur-MMs) and C6-Mixed Micelles (C6-MMs)

#### 2.1.1. Critical Micelle Concentration (CMC)

In order to obtain stable micelles, the CMC of Solutol HS 15 and PEG-DSPE mixtures at different ratios were compared. The results showed that the CMC values of micelles with a Solutol HS 15 and PEG-DSPE ratio of 3:2 (*w*/*w*) were the lowest, indicating that the micelles were the most stable at a ratio of 3:2 [12]. Hence, 3:2 was chosen as the weight ratio of Solutol HS 15 and PEG-DSPE (3:2) for the fabrication of the micelles (Table 1).

#### 2.1.2. Morphology, Particle Size, Zeta Potential, Drug Loading, and Entrapment Efficiency

**Figure 1:**
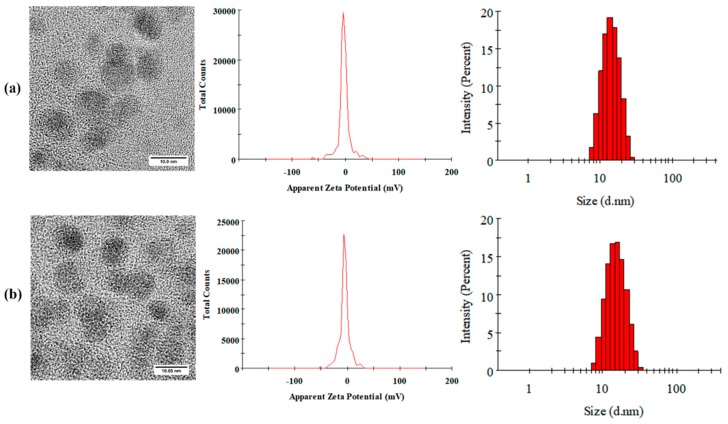
Morphology, zeta potential, and size distribution images of (**a**) curcumin mixed micelles Cur-MMs and (**b**) C6-mixed micelles (C6-MMs).

The morphologies of Cur-MMs and C6-MMs were examined using transmission electron microscopy (TEM). As seen in Figure 1, the micelles exhibited almost spherical and uniform shapes, with dark solid spheres. The mean particle size was 13.49 ± 0.18 nm (with a negative zeta potential value of −4.65 ± 0.30 mV) for Cur-MMs and 14.32 ± 0.47 nm (with a zeta potential value of −4.86 ± 0.42 mV) for C6-MMs. With respect to size distribution, all polydispersity index (PDI) values were less than 0.3, which indicates that the micelles were homogeneously distributed. Table 2 shows that the encapsulation efficiency was 97.28% ± 2.44% for Cur-MMs and 91.10% ± 4.56% for C6-MMs. Moreover, the degrees of drug loading were 2.28% ± 0.06% and 0.04% ± 0.003% for Cur-MMs and C6-MMs, respectively.

#### 2.1.3. Crystalline Form of Cur-MMs

**Figure 2:**
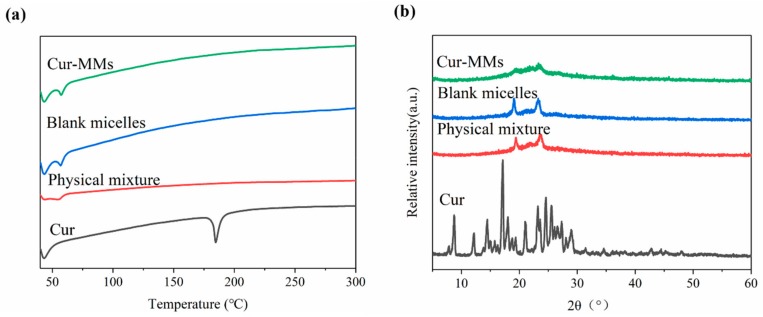
(**a**) Differential scanning calorimetry (DSC) profiles of Cur, the physical mixture, blank micelle, and Cur-MMs. (**b**) Polycrystalline X-ray diffraction (PXRD) of curcumin (Cur), physical mixture, blank micelle, and Cur-MMs.

Differential scanning calorimetry (DSC) and polycrystalline X-ray diffraction (PXRD) measurements were carried out to characterize the physical status of the Cur present in Cur-MMs. As shown in the DSC patterns in Figure 2a, a single sharp endothermic melting peak was found for Cur at 178.8 °C, as its melting indicated its crystalline nature. However, no curcumin peak was observed in the physical mixture, blank micelle, or Cur-MMs. These data demonstrate that curcumin is dispersed in a non-crystalline state in the carrier. Typically, there is no melting point peak found for the drug in the physical mixture. This unexpected result might be explained by the fact that Cur in the physical mixture dissolved in the melted Solutol HS 15 before it reached its melting point [13]. As shown in Figure 2b, the typical diffraction peaks of Cur were visible between 5° and 30°. However, no trace of the typical crystalline peaks of Cur was found in the other groups. This result is consistent with that of DSC, indicating that Cur was in an amorphous state in the Cur-MMs mixed micelles.

### 2.2. Cellular Uptake Studies

**Figure 3:**
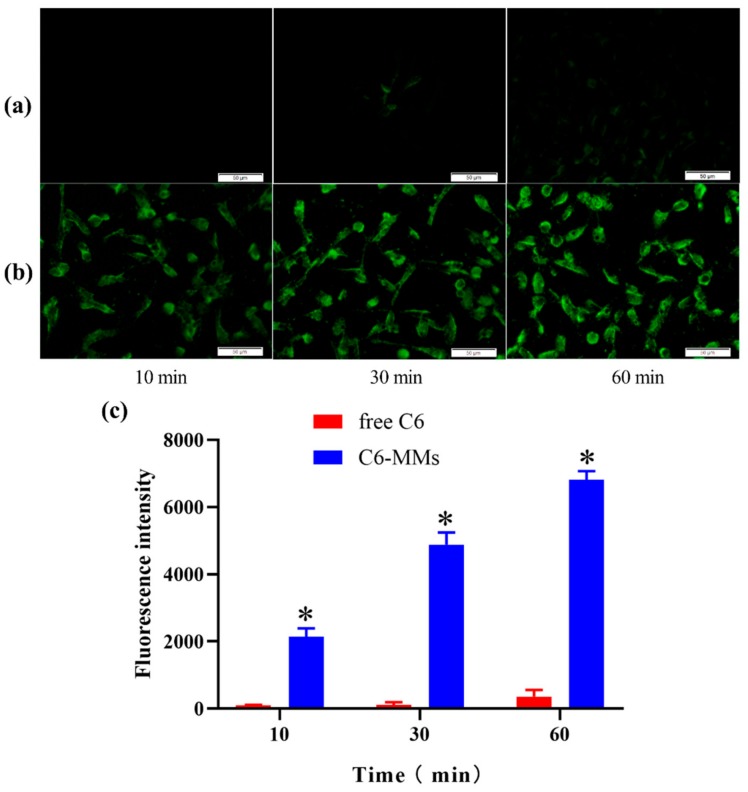
Cellular uptake of coumarin-6 (C6) by human corneal epithelial cells (HCECs). (**a**) Fluorescence inverted microscope observations of the uptake characteristics of free C6. (**b**) Fluorescence inverted microscope observations of the uptake characteristics of C6-MMs. (**c**) Uptake of free C6 and C6-MMs into HCECs according to flow cytometry (* *p* < 0.05 when compared with free C6).

Compared with the free C6, there was a more obvious green fluorescence when the human corneal epithelial cells (HCECs) were incubated with C6-MMs. These results show that the PEG-DSPE/Solutol HS 15 mixed micelles were rapidly and time-dependently taken up by the HCECs (Figure 3). The results imply that increasing the interaction time between the C6-MMs and HCECs increases the absorption of C6-MMs.

### 2.3. Characterization of Curcumin Mixed Micelle In Situ Gelling System (Cur-MM-ISG)

#### 2.3.1. Particle Size, Osmotic Pressure, pH, and Transmittance of Cur-MM-ISG

The particle size of Cur-MM-ISG was 15.51 ± 0.15 nm. Furthermore, the PDI value was 0.19 ± 0.03, indicating that the micelles in Cur-MM-ISG maintained a complete structure. The osmotic pressure was 321 mosmol/kg and the pH was 6.8 for Cur-MM-ISG. Given that an increase in the viscosity of ophthalmic formulations often causes blurred vision, it is essential to evaluate the transmittance of ophthalmic preparations. The transmittance (T%) of simulated tear fluids (STF) was 100.07% ± 0.06%. The transmittance (T%) of the mixture of the Cur-MM-ISG and STF (40:7, *v*/*v*) was 98.90% ± 0.00%.

#### 2.3.2. Test for Rheological and Gelling Ability

**Figure 4:**
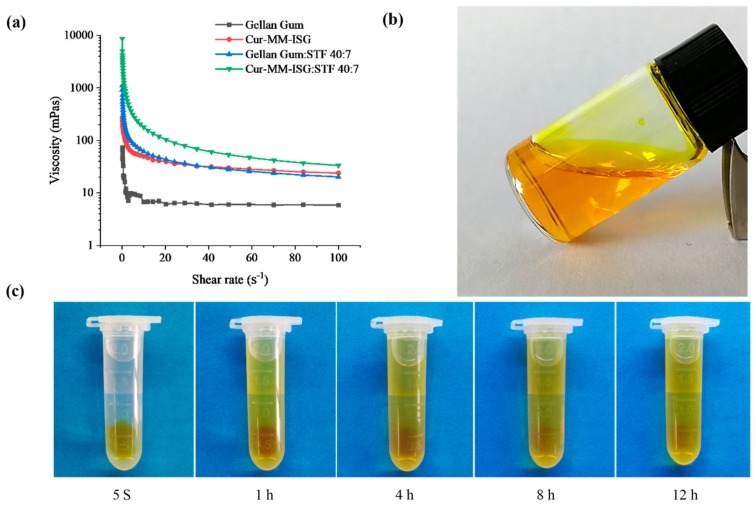
(**a**) Rheogram showing the viscosity of gellan gum (■), curcumin-mixed micelle in situ gelling system (Cur-MM-ISG) (●), mixture of gellan gum and simulated tear fluids (STF) (40:7 *v*:*v*) (▲), mixture of Cur-MM-ISG, and STF (40:7 *v*:*v*) (▼). All formulations were measured at 34 °C. (**b**) Picture of Cur-MM-ISG at 25 °C. (**c**) Test for the gelling ability of Cur-MM-ISG at 34 °C.

Figure 4a shows the rheology of the different formulations. All formulations included shear thinning and exhibited pseudoplastic rheology (i.e., a decrease in the viscosity with an increase in angular velocity). The rheological behaviors of gellan gum were not affected by the addition of Cur-MMs. The gelling capacity of Cur-MM-ISG was evaluated to identify the formulation suitable for use with in situ gelling systems. Cur-MM-ISG mixed with STF could form a gel immediately, while the Cur-MM-ISG formed a gel that could last for 12 h (Figure 4c).

#### 2.3.3. In Vitro Drug Release Studies

**Figure 5:**
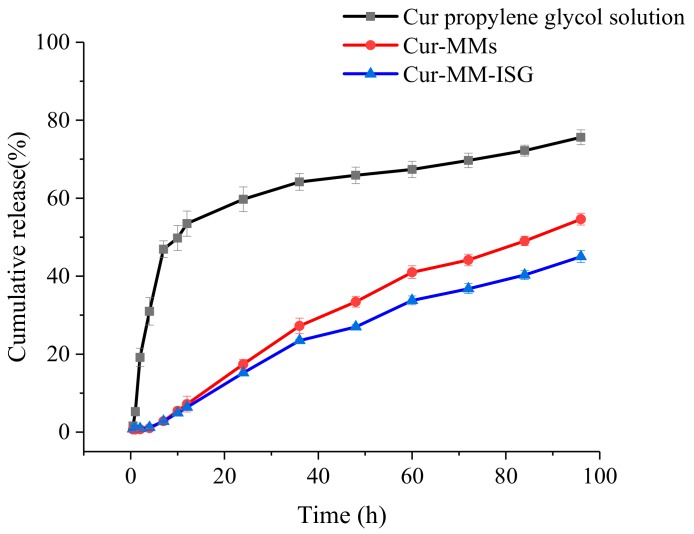
Profiles of the cumulative percentage release of Cur from the propylene glycol solution (■), Cur-MMs (●), and Cur-MM-ISG (▲) at predetermined periods in PBS containing 5% Tween 80.

The in vitro release of Cur from different formulations was studied using the dialysis method at 34 °C. As described in Figure 5, over 19.15% of the Cur was dissolved in the release medium from the propylene glycol solution within 2 h and nearly 59.74% of the drug was released after 24 h. However, the Cur-MMs presented a sustained release profile within 24 h and 17.40% of the drugs were received in the phosphate buffer saline (PBS). The release profile of Cur-MMs and Cur-MM-ISG followed fist-order kinetics, with a regression factor of 0.996 and 0.993, respectively [14]. The statistical analysis of release data was performed by comparing the similarity factor (f_2_). The similarity factor between the Cur-MMs and Cur-MM-ISG was 61.2. An f_2_ value between 50 and 100 suggests that two dissolution profiles are similar and indicates a point-to-point difference of 10% or less [15].

#### 2.3.4. Chemical Stability

**Figure 6:**
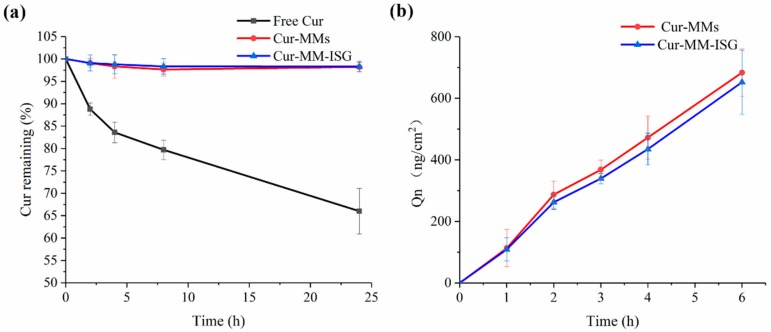
(**a**) Stability of free Cur (■), Cur-MMs (●), and Cur-MM-ISG (▲) in pH 6.8 Tris(hydroxymethyl) aminomethane (Tris)-HCl buffer at 25 °C. (**b**) Ex vivo corneal penetration curves of Cur-MMs (●) and Cur-MM-ISG (▲) at 34 °C.

After incubation in a Tris-HCl buffer (pH 6.8), 34% of free curcumin degraded after 24 h at 25 °C (Figure 6a). In contrast to free curcumin, Cur-MMs and Cur-MM-ISG showed enhanced Cur chemical stability in the solution, with only 1.4% and 1.2% curcumin degradation detected, respectively.

### 2.4. Ex Vivo Penetration Study

The ex vivo corneal penetration curves of the Cur-MMs and Cur-MM-ISG are shown in Figure 6b. The cumulative drug permeation amounts (Q_n_, ng/cm^2^) of the Cur-MMs and Cur-MM-ISG were 683.48 ± 76.84 and 652.20 ± 104.15 ng/cm^2^ at 6 h, respectively. There was no significant difference between the two groups (*p* > 0.05). In contrast, we did not detect curcumin in the receiving solution for the free curcumin group. The results showed that the Cur-MM-ISG enhanced the penetration of the curcumin across the cornea.

### 2.5. Ocular Irritation Test

**Figure 7:**
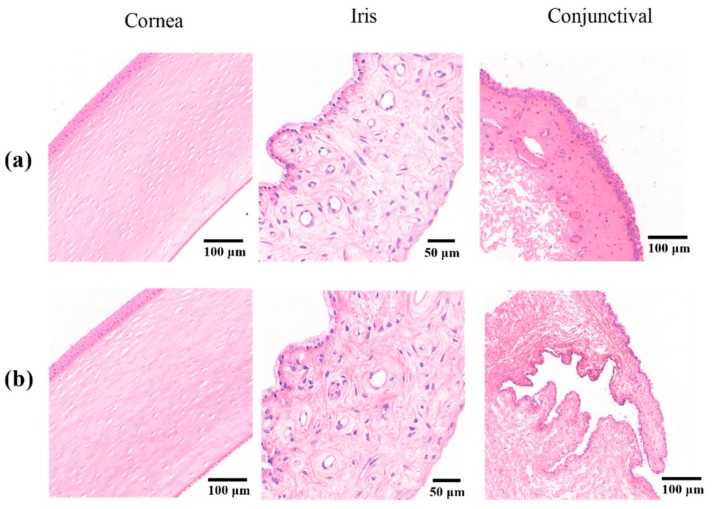
Histopathological sections of the cornea (left), iris (middle), and conjunctiva (right) treated with (**a**) saline solution or (**b**) Cur-MM-ISG. Original magnification: 150× (cornea), 400× (iris), and 100× (conjunctiva).

Figure 7 shows histopathological microscopy images of the three tissues (cornea, iris, and conjunctiva) treated with Cur-MM-ISG. Compared with the control group, the results from the ocular tolerability tests showed no ocular toxicity or irritation of the external ocular tissues in rabbit eyes at any time point after the instillation of Cur-MM-ISG. The results of histopathological examinations showed no changes in the morphologies of the cornea, iris, and conjunctiva. Furthermore, fluorescein staining did not suggest any corneal epithelial defects. Thus, the new nanocomposite hydrogel did not produce eye irritation, making it suitable for ocular applications.

### 2.6. Pre-Corneal Retention Study

**Figure 8:**
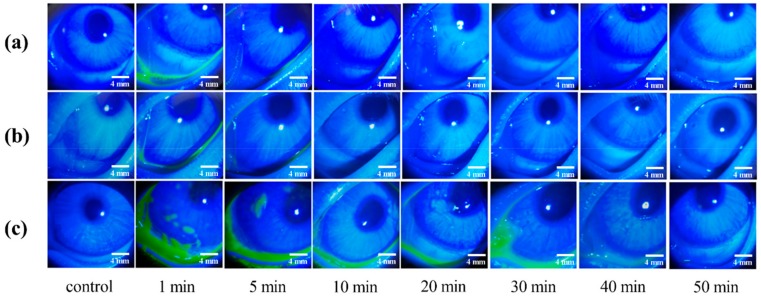
In vivo fluorescence imaging of (**a**) 0.1% sodium fluorescein solution, (**b**) Cur-MMs, and (**c**) Cur-MM-ISG at various time points post-dropping of the three formulations.

The green fluorescence disappearance time was taken as the retention time for the comparison of the different preparations. The retention time of 0.1% sodium fluorescein solution and Cur-MMs was 10 min, while the retention time of Cur-MM-ISG was 50 min (Figure 8). These results indicate that gellan gum increased the eye retention time of the Cur-MMs fivefold.

### 2.7. Corneal Permeation Studies in Rabbit Eyes

**Figure 9:**
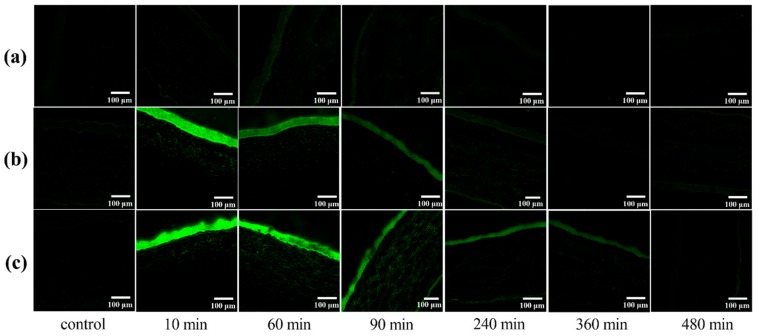
Confocal laser scanning microscopy (CLSM) of rabbit corneal tissues after the in vivo corneal permeation of free (**a**) C6, (**b**) C6-mixed micelles (C6-MMs), and (**c**) C6 mixed in situ micelle gel (C6-MM-ISG) at various times.

To evaluate the capacity of different preparations to improve in vivo corneal permeation, confocal laser scanning microscopy (CLSM) was employed to investigate the transcorneal penetration behavior. The corneal images observed at various time points after administration are shown in Figure 9. There was very weak fluorescence for free C6 after 30 min of administration, which is consistent with the literature [16]. The C6-MMs absorbed rapidly after administration and the obvious green fluorescent signals primarily focused on the corneal epithelium for 10 min. Moreover, a weak green fluorescence was observed in the corneal stroma and the endothelium. The fluorescence intensity of the corneal epithelium and stroma decreased significantly and disappeared at 240 min. However, the C6 mixed in situ micelle gel (C6-MM-ISG) exhibited a stronger fluorescence signal and a deeper transmission intensity into the entire cornea than C6-MMs. For C6-MM-ISG, the green fluorescence was clearly observed in the stroma at 90 min. At 360 min, the corneal epithelium was also well stained. These results sustain the hypothesis that Cur can penetrate through the cornea more efficiently after being loaded into mixed micelles and gellan gum gels.

## 3. Discussion

Curcumin is a naturally hydrophobic polyphenol with potential pharmacological benefits. The experimental and clinical data obtained so far indicate that orally-administered curcumin is well-tolerated and has been shown to be safe in humans [17]. However, the poor aqueous solubility and low bioavailability of curcumin are limiting factors for the formulation of solutions intended for ocular administration. Thus, the development of micelles able to enhance the solubility of hydrophobic drugs and improve eye surface penetrability is of great interest to ophthalmologists.

In order to improve the solubility, stability, and corneal permeability of curcumin, a novel ophthalmic gel-forming solution based on PEG-DSPE/Solutol HS 15 mixed micelle in situ gel was constructed in this study. When Cur was encapsulated in mixed micelles, the solubility of curcumin was enhanced to 3.96 mM, compared to 30 nM [18,19] in an aqueous solution. The chemical stability test showed that Cur-MMs enhance curcumin chemical stability, with only 1.4% curcumin degradation detected within 24 h, while 34% of free curcumin degraded in the Tris-HCl buffer (pH 6.8). The ex vivo penetration study showed that the Cur-MMs enhanced the penetration of the curcumin across the cornea. In general, smaller particles display higher cellular uptake and a greater ability to across the biobarrier than larger particles [20]. Moreover, negatively-charged micelles diffuse more easily in the corneal epithelium or in the corneal stroma than positively charged colloidal carriers [21]. The mean particle size was 13.49 nm, with a negative zeta potential value of −4.65 mV for Cur-MMs, which might be the reason why Cur-MMs increased the corneal permeability of curcumin.

In order to prolong the ocular retention time of Cur-MMs, the Cur-MM and gellan gum solutions were mixed in order to prepare the Cur-MM-ISG. The results demonstrated that the osmotic pressure of the formulation was 321 mOsmol/kg and the pH was 6.8 for Cur-MM-ISG, which is close to that of human tears [22,23]. Moreover, no ocular irritation was observed in the external ocular tissues of rabbit eyes after the instillation of Cur-MM-ISG. The histopathological results showed no changes in the morphologies of the cornea, iris, or conjunctiva. The Cur-MM-ISG was of a shear thinning-type and exhibited pseudoplastic rheology, a decrease in viscosity, and an increase in angular velocity. For ophthalmic administration, pseudoplastic fluid could increase the viscosity of the drug without affecting the blinking of the patient. Furthermore, in vivo experiments showed that Cur-MM-ISG increased the eye retention time of the Cur-MMs, possibly due to the formation of a gel network by gellan gum under the influence of cations from tears [24]. These results suggest that curcumin can penetrate through the cornea more efficiency after being loaded into PEG-DSPE/Solutol HS 15 mixed micelles and gellan gum gels.

## 4. Materials and Methods

### 4.1. Materials

The PEG2000-DSPE (Corden, Liestal, Swiztherland), Solutol HS 15 (BASF, Ludwigshafen, Germany), Cur, C6, deacetylated gellan gum, and Tween 80 were purchased from Sigma-Aldrich (Saint Louis, MO., USA). Tris(hydroxymethyl) aminomethane (Tris)-HCl buffer (pH 6.8 and 7.0) and mannitol were purchased from Beijing Baiardi Biotechnology Co. Ltd. (Beijing, China). Pyrene and PBS (PH 7.4) were products of Shanghai McLean Biochemical Technology Co. Ltd. (Beijing, China) and Beijing Solarbio Science & Technology Co. Ltd. (Beijing, China), respectively. HCECs were purchased from FuDan IBS Cell Center (Shanghai, China). Dulbecco’s Modified Eagle Medium (DMEM), fetal calf serum (FBS), and penicillin-streptomycin solution were purchased from Corning (New York, NY., USA). Bovine insulin and epithelial growth factor (EGF) were products of Cyagen Biosciences (Santa Clara, CA., USA). FAS eye fixer was purchased from Servicebio technology Co. Ltd. (Wuhan, China). Other chemicals and reagents were of analytical grade. New Zealand albino rabbits weighing 2–2.5 kg, devoid of any signs of ocular inflammation or gross abnormalities, were purchased from Beijing Jinmuyang Laboratory Animal Breeding Co. Ltd. (Beijing, China) ([license no: SCXK (Beijing) 2015-0005].

### 4.2. Preparation of Cur-MMs and C6-MMs

The mixture of Cur (6 mg), Solutol HS 15 (150 mg), and PEG-DSPE (100 mg) was melted in ethanol and vortexed for 5 min. Then, the solvent was evaporated under reduced pressure at 37 °C. The film was then hydrated with 2 ml Tris-HCl buffer (pH 7.0) and stirred for 1 h at 37 °C. Thereafter, the mixture was centrifuged (Eppendorf 5424R centrifuge, Eppendorf AG, Hamburg, Germany) at 12,000 rpm for 10 min and filtered through a 0.22 μm membrane to remove any undissolved drugs. To observe the drug distribution behavior of the PEG-DSPE/Solutol HS 15 mixed micelles, C6 was loaded into the micelles following the same procedures as described above.

### 4.3. Characterization of Cur-MMs and C6-MMs

#### 4.3.1. CMC

The CMC was measured using fluorescence spectroscopy, with pyrene as a fluorescent probe [25]. The Solutol HS 15 and PEG-DSPE were weighed in different proportions and diluted to yield concentrations in the range of 0.06–2000 µg·mL^−1^. Each polymer solution was transferred to a vial containing pyrene. The intensities of the I_1_ (373 nm) and I_3_ (384 nm) vibronic bands were evaluated and the ratio of these intensities were plotted against the logarithm of the concentration of each sample. The CMC was taken as the intersection of two regression lines calculated from the linear portions of the graphs [26].

#### 4.3.2. Morphology, Particle Size, and Zeta Potential

The morphologies of the Cur-MMs and C6-MMs were acquired using TEM (JEM-1200EX JEOL, Tokyo, Japan). The size distribution and zeta potential were evaluated with Zetasizer Nano ZS (Malvern Co., Malvern, UK) by diluting the micelles with the appropriate amount of pH 7.0 Tris-HCl buffer.

#### 4.3.3. Drug loading (DL) and encapsulation efficiency (EE)

The Cur-MMs sample (or C6-MMs) (0.1 mL) was dissolved in 10 mL of methanol. In order to completely break up the micelles, the mixture was placed in an ultrasonic bath for 10 min. The solution was filtered through 0.22 μm membrane filters and subjected to high-performance liquid chromatography (HPLC) using an Agilent Eclipse XBD C18 reverse-phase column (4.6 mm × 250 mm, 5 μm). The mobile phase consisted of a mixture of acetonitrile and water (52:48, *v*/*v*). The flow rate was 1.0 mL/min at 25 °C, and the detection wavelength was 430 nm. The DL and EE were calculated as shown below Equations (1) and (2) [27,28]:

(1) $$\mathrm{DL}\%=\frac{\mathrm{weight}\;\mathrm{of}\;\mathrm{drug}\;\mathrm{in}\;\mathrm{micelles}}{\mathrm{weight}\;\mathrm{of}\;\mathrm{feeding}\;\mathrm{carriers}\;\mathrm{and}\;\mathrm{drug}}\times 100\%$$

(2) $$\mathrm{EE}\%=\frac{\mathrm{weight}\;\mathrm{of}\;\mathrm{drug}\;\mathrm{in}\;\mathrm{micelles}}{\mathrm{weight}\;\mathrm{of}\;\mathrm{feeding}\;\mathrm{drug}}\times 100\%$$

#### 4.3.4. DSC and PXRD

Thermal analysis was carried out using a DSC calorimeter (Mettler Toledo, Schwerzenbach, Switzerland). Samples were placed in aluminum pans and heated at a scanning rate of 10 K/min under nitrogen from 40 °C to 300 °C [29]. The PXRD analysis was performed at room temperature with a DX-2700 micro-diffractometer (Aolong Radiative Instrument Group Co. Ltd., Dandong, China). Data were recorded under graphite monochromatized Cu Kα radiation over the 2θ range from 5° to 60° at 40 kV and 50 mA [30].

### 4.4. Cellular Uptake Studies

The HCECs were incubated at a 37 °C and 5% CO_2_ atmosphere in complete DMEM containing 10 % FBS, 100 U/mL penicillin, 100 μg/mL streptomycin, 10 ng/mL EGF, and 5 μg/mL bovine insulin. The HCECs were seeded at a density of 5 × 10^5^ cells/well in 12-well plates. The culture medium was thereafter discarded and the cells were washed twice with PBS. Then, the cells were incubated with test solutions at 37 °C for 10, 30, and 60 min, followed by washing thrice with PBS to remove the non-intracellular drug. The HCECs were then photographed with a fluorescence inverted microscope (Olympus IX53IX53, Tokyo, Japan). The mean intracellular fluorescence intensity was measured flow cytometrically at an excitation wavelength of 488 nm. The results are reported as the mean of the distribution of the cell fluorescence intensity obtained by measuring approximately 10,000 cells [31].

### 4.5. Cur-MM-ISG Preparation

The in situ gelling system was prepared by dispersing PEG-DSPE/Solutol HS 15 mixed micelles into a gel solution. Gellan gum (0.5 g) was dispersed in 50 mL deionized water and heated at 90 °C for 2 h until completely dissolved and then restored to room temperature [32]. The Cur-MMs and gellan gum solutions were mixed at a ratio of 1:1 (*v*:*v*), and a suitable amount of mannitol was added to adjust the osmotic pressure.

### 4.6. Characterization of Cur-MM-ISG

#### 4.6.1. Particle Size, Osmotic Pressure, pH, and Transmittance

Size distribution was evaluated with a Zetasizer Nano ZS (Malvern Co. UK) by diluting the Cur-MM-ISG with appropriate amount of Tris-HCl buffer. The osmotic pressure of the formulation was evaluated using a freezing point osmotic pressure meter (YASN Osmolab One, Beijing, China). The pH was measured using a pH meter (Sartorius PB-10, Gottingen, Germany). Moreover, the transparency of Cur-MM-ISG was assessed by detecting the percentage transmittance (%T) at 700 nm [33] using a UV-Vis spectrophotometer (Beckman Coulter DU-800 UV/Vis Spectrophotometer, Fullerton, CA, United States).

#### 4.6.2. Test for Rheological Studies and Gelling Ability

The viscosity of the prepared formulation was analyzed at different angular velocities at 34 °C using a rotational viscometer (Haake MARS 40, Shanghai, China). The cone angle was 1° and the diameter of the rotating cone was 35 mm. Evaluations were conducted in triplicate at a series of shear rates (0.1–100/s) and the corresponding viscosity values were recorded [34]. Cur-MM-ISG (100 µl) was added to 2 mL of STF, which contained 6.78 g of NaCl, 2.18 g of NaHCO_3_, 0.084 g of CaCl_2_, and 1.38 g of KCl per liter of ultrapure water. The phase transition of the solution to a viscous gel was then observed [35].

#### 4.6.3. In Vitro Release Studies

To evaluate the release kinetics of Cur from the prepared formulations, a release study was carried out using a dialysis membrane with molecular weight cut-off of 8000 to 14,000 [36]. Each drug-loaded formulation (including 0.5 mg curcumin and STF (40:7, *v*/*v*)) was successively put into the dialysis bag and then immersed in the receiving vessels containing 60 mL of the release medium. At the predetermined periods, 2 mL of release medium was withdrawn and replaced with an equal volume of fresh medium at each sampling time. The amount of Cur was determined at 430 nm using HPLC (Agilent 1200, Agilent Technologies Corp., Santa Clara, CA., USA). A Cur solution dissolved in propylene glycol (control group) was evaluated using the same dissolution condition. All release experiments were performed at 34 °C.

#### 4.6.4. Chemical Stability

Studies on the stability of curcumin used the previously reported method [37]. Aliquots of 50 μL of Cur dissolved in ethanol ([Cur] = 1 mg/mL) or Cur-MMs-ISG were added to 950 μL portions of pH 6.8 Tris-HCl buffer. Samples were incubated at 25 °C for the indicated times. After incubation, the mixture was diluted by 1 mL of ethanol. Then, the solution was filtered through a 0.22 μm membrane and analyzed by HPLC (Agilent 1200, Agilent Technologies Corp., USA).

### 4.7. Ex Vivo Penetration Study

The corneal permeation evaluation consulted the paper of Gao et al. [38]. The rabbits were sacrificed by injecting air into the marginal ear vein. The corneas were dissected with a 2 mm scleral ring and the cornea was immediately mounted over the modified Franz-type vertical diffusion chambers. STF and the prepared formulations containing 400 μg curcumin were added to the compartment at a volume ratio of 40:7. The solution in the receiver was a fresh glutathione bicarbonate Ringer’s solution (GBR) with 5% Tween-80 (*v*/*v*). At each time interval of 1 h, a 1 mL sample was withdrawn from the receptor chamber. The curcumin concentration was determined by HPLC (Agilent 1200, Agilent Technologies Corp., USA). All formulations were measured at 34 °C.

### 4.8. Ocular Irritation Test for Cur-MMs-ISG

New Zealand albino rabbits were housed in standard cages in a light-controlled room at 19 ± 1 °C and a relative humidity of 50% ± 5% and were fed a standard pellet diet with water ad libitum. All tests complied with the Guide for the Care and Use of Laboratory Animals, Institute of Laboratory Animal Resources, and were approved by the Institutional Animal Care and Use Committee of the Beijing University of Chinese Medicine.

The eye irritancy potential of Cur-MM-ISG was carefully determined with a modified Draize test using a slit-lamp microscope [39], adopting a self-contrast method in which the right eye was directly given 40 μL Cur-MM-ISG and the left eye was given an equivalent amount of isotonic sodium chloride solution. Ocular conditions were recorded every day and at 1, 2, 4, 24, 48, and 72 h after the last administration. The cornea, iris, and conjunctiva were fixed with an eye fixer, paraffin-embedded, sectioned, stained with hematoxylin-eosin (H&E), and scanned with a Panoramic Desk (3D Histotech Panoramic digital slide scanner, Budapest, Hungary).

### 4.9. Pre-Corneal Retention Study

The prepared formulations (Cur-MMs and Cur-MM-ISG) were infused into the conjunctival sac and 0.1% (*w*/*v*) sodium fluorescein was added to the preparation in order to make it fluorescent [40]. Each group was photographed after 1, 5, 10, 20, 30, 40, and 50 min under the cobalt blue light of a YZ3 slit lamp microscope (Suzhou Liuliu Vision Technology Co. Ltd., Suzhou, China).

### 4.10. In Vivo Corneal Permeation Experiment

To investigate the in vivo transcorneal behavior of the drug within the rabbit eye, a confocal laser scanning microscope (NIKON Eclipse Ti, Japan) was employed [41]. The preparation (40 µL) was dripped into the eyes of each animal. Control animals received an equivalent volume of normal saline. At fixed time intervals (10, 60, 90, 240, 360, and 480 min), the rabbits were euthanized via air embolism and their eyes were instantly removed. The corneas were isolated and rinsed in physiological saline to remove the adhesive material and sliced vertically along the sagittal plane using a cryostat microtome (Thermo NX50, Waltham, MA. USA). The corneal slices were observed and recorded via confocal laser scanning microscopy.

### 4.11. Statistical Analysis

All experiments were performed at least in triplicate and the data expressed as the mean ± standard deviation (SD). The statistical analysis was performed by a One-Way ANOVA and Fisher’s least significant difference (LSD) test using the SPSS 17.0 software (SPSS, Inc., Chicago, IL, USA). A *p* value < 0.05 was considered statistically significant.

## 5. Conclusions

A novel ophthalmic gel-forming solution based on PEG-DSPE/Solutol HS 15 mixed micelles and gellan gum for ophthalmic delivery was successfully developed. Cur-MM-ISG was transparent and exerted no irritation on ocular tissues, which improved the solubility, stability, and corneal permeability of curcumin significantly. These findings indicate that biocompatible Cur-MM-ISG has great potential in efficient ophthalmic drug therapy.

## Figures and Tables

**Table 1:** Critical micelle concentration (CMC) for polyoxyethylene esters of 12-hydroxystearic acid (Solutol HS 15), 1,2-distearoyl-sn-glycero-3-phosphoethanolamine-N-[methoxy(polyethylene glycol)-2000] (PEG-DSPE), and varying ratios Solutol HS 15 to PEG-DSPE (wt% ratio).

Ratio	Solutol HS 15	4:1	3:2	2:3	1:4	PEG-DSPE
CMC (μg/mL)	66.06 ± 5.25	58.85 ± 3.60	41.66 ± 4.46	64.58 ± 5.74	93.29 ± 5.60	104.69 ± 9.61

**Table 2:** Particle size (PS), polydispersity index (PDI), zeta potential (ZP), drug loading (DL), and encapsulation efficiency (EE) of Cur-MMs and C6-MMs. Values are the mean ± SD (*n* = 3).

Sample	PS (nm ± SD)	PDI	ZP (mV ± SD)	DL% (±SD)	EE% (±SD)
Cur-MMs	13.49 ± 0.18	0.07 ± 0.004	−4.65 ± 0.30	2.28 ± 0.06%	97.28 ± 2.44%
C6-MMs	14.32 ± 0.47	0.24 ± 0.03	−4.86 ± 0.42	0.04 ± 0.003%	91.10 ± 4.56%
Blank MMs	13.33 ± 0.13	0.08 ± 0.02	−4.79 ± 1.31	-	-
